# Virtual Screening for FDA-Approved Drugs That Selectively Inhibit Arginase Type 1 and 2

**DOI:** 10.3390/molecules27165134

**Published:** 2022-08-12

**Authors:** Trishna Saha Detroja, Abraham O. Samson

**Affiliations:** The Azrieli Faculty of Medicine, Bar-Ilan University, Safed 1311502, Israel

**Keywords:** virtual screening, molecular docking, ARG1, ARG2, arginase, vina, FDA

## Abstract

Arginases are often overexpressed in human diseases, and they are an important target for developing anti-aging and antineoplastic drugs. Arginase type 1 (ARG1) is a cytosolic enzyme, and arginase type 2 (ARG2) is a mitochondrial one. In this study, a dataset containing 2115-FDA-approved drug molecules is virtually screened for potential arginase binding using molecular docking against several ARG1 and ARG2 structures. The potential arginase ligands are classified into three categories: (1) Non-selective, (2) ARG1 selective, and (3) ARG2 selective. The evaluated potential arginase ligands are then compared with their clinical use. Remarkably, half of the top 30 potential drugs are used clinically to lower blood pressure and treat cancer, infection, kidney disease, and Parkinson’s disease thus partially validating our virtual screen. Most notable are the antihypertensive drugs candesartan, irbesartan, indapamide, and amiloride, the antiemetic rolapitant, the anti-angina ivabradine, and the antidiabetic metformin which have minimal side effects. The partial validation also favors the idea that the other half of the top 30 potential drugs could be used in therapeutic settings. The three categories greatly expand the selectivity of arginase inhibition.

## 1. Introduction

**Figure 1:**
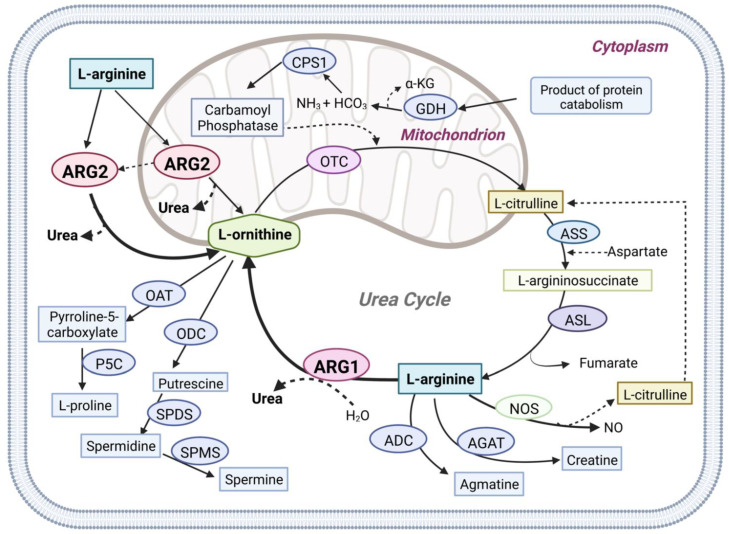
Graphical overview of Arginase1 (ARG1) and Arginase2 (ARG2) mediated L-arginine metabolism in physiologic pathways. Arginase1 (ARG1), nitric oxide synthases (NOS), arginine decarboxylase (ADC), and L-arginine: glycine amidinotransferase (AGAT) use L-arginine as the primary metabolic substrate. Arginase1, the final enzyme of the urea cycle, cleaves L-arginine to form urea and ornithine in the cytoplasm. Arginase2 (ARG2) is also involved in the urea cycle L-arginine metabolism in both the cytoplasm and mitochondria. N-acetyl glutamate synthase (NAGS), carbamoyl phosphate synthase (CPS1), ornithine transcarbamylase (OTC), argininosuccinate synthetase (ASS), argininosuccinate lyase (ASL), glutamate dehydrogenase (GDH), ornithine aminotransferase (OAT), ornithine decarboxylase (ODC), Δ1-pyrroline-5-carboxylate synthase (P5C), spermidine synthase (SPDS), spermine synthase (SPMS).

Arginase (E.C. 3.5.3.1) is a ureohydrolase homotrimeric binuclear manganese metalloenzyme that hydrolyses L-arginine into L-ornithine and urea [1,2] (Figure 1). In ureotelic animals, this enzyme is primarily involved in the elimination of excess ammonia through the urea cycle [3]. Interestingly, overexpression of arginase is involved in diseases such as cancer, cardiovascular, diabetes, asthma, neurodegenerative disease, and infectious disease [4,5,6,7,8]. In mammals two distinct isoforms of the enzyme exist, namely arginase type 1 (ARG1) and arginase type 2 (ARG2), which are encoded by different genes, and share approximately 60% sequence homology [9]. The isoenzymes differ in tissue distribution, intracellular location, and immunochemical characteristics [9]. ARG1 is a cytosolic enzyme expressed predominantly in the liver, where it plays a key role in urea synthesis [10]. ARG2 is a mitochondrial enzyme that is expressed in most tissues, with high levels found in the kidney and prostate, but also in blood vessels, intestines, red blood cells, and immune cells. Recent evidence suggests that ARG2 can translocate from the mitochondrion into the cytosol leading to an increase in its activity [11].

**Figure 2:**
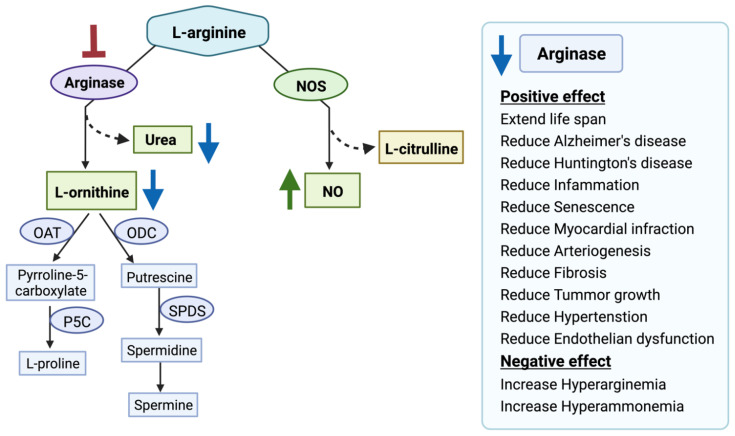
Arginase inhibition and its clinical relevance. Arginase inhibition results in increased nitric oxide (NO) production and decreased L-ornithine and urea production. Nitric oxide synthases (NOS), ornithine aminotransferase (OAT), ornithine decarboxylase (ODC), Δ1-pyrroline-5-carboxylate synthase (P5C), spermidine synthase (SPDS), spermine synthase (SPMS). The red blunt arrow shows inhibition, the green arrow represents elevated levels of the substances; blue arrows designate reduced ones.

Arginase competes with several key enzymes for its substrate L-arginine, notably with nitric oxide synthetase (NOS), arginine decarboxylase (ADC), glycine amidinotransferase (AGAT), arginine deiminase, etc [12]. Over-expression of ARG1 or ARG2 disrupts L-arginine homeostasis which leads to metabolic disorder-related diseases, Alzheimer’s disease (AD), cancer, etc [13,14,15,16]. Studies have shown that ARG2 is over-expressed in AD patients’ brains compared to the control brain [17]. ARG2 over-expression leads to L-arginine depletion and increased production of urea [18]. Handley et al., observed increased urea production and hypothesized that an altered urea cycle could be a common feature of Huntington’s disease [19]. Arginase overexpression is also related to increased proline and polyamines synthesis via the arginase-ornithine pathway leading to fibrosis, tumor growth, and invasion [20,21]. Arginase overexpression also reduces the bioavailability of NO (nitric oxide) which leads to increased blood pressure and endothelial dysfunction [22,23,24,25]. Furthermore, arginase overexpression impairs T-cell function leading to immune dysfunction-mediated cancer progression [26,27]. Moreover, it also interacts with different signaling pathways such as MAPK, STAT3, and AKT-mTOR involved in tumorigenesis [8,15,28,29]. Another recent study has shown that ARG2 has involved in obesity-related metabolic dysfunction [30]. Several other studies have shown that ARG2 plays a critical role in advanced inflammation, oxidative stress, and senescence [31,32,33]. Finally, we have proposed arginase as a potential target in the treatment of AD [34,35,36]. Hence, arginases present a promising target in the treatment of various diseases (Figure 2).

There are a few potent and selective inhibitors available that regulate the balance between NOS and the arginase pathway by controlling arginase activity. For example, the arginase inhibitors N-omega-hydroxy-L-arginine (NOHA), nor-N-omega-hydroxy-L-arginine (nor-NOHA), 2(S)-amino-6-boronohexanoic acid (ABH), S-(2-boronoethyl)-L-cysteine (BEC) are currently in a pre-clinical trial [15,37]. Moreover, plant-derived compounds such as polyphenols “chlorogenic acid” [38], glycoside derivative “Piceatannol-3′-O-β-D-glucopyranoside” [39], flavonoids “(2S)-5,2′5′-trihydroxy-7,8-dimethoxy” [40], and cinnamide derivatives [41] are also investigated for developing potent arginase inhibitor. In addition, arginase inhibition using L-norvaline reverses neurodegeneration in a murine model of Alzheimer’s disease [42]. However, one potential concern is the lack of selectivity of the drugs for ARG1 and ARG2. ARG1 expression in macrophages is critical for tissue repair [43]. Additionally, ARG1 inhibition leads to episodic hyperarginemia and hyperammonemia [44]. ARG1 inhibition is detrimental and can cause death if left untreated [45]. Moreover, ARG1 knockout is lethal in mice [46]. In contrast, selective ARG2 inhibition prevents atherosclerosis [32], delays vascular aging [47], suppresses insulin resistance [48], ameliorates diabetic renal injury [49], protects against hypoxia-induced pulmonary hypertension [50], and prevents oxidative stress [31]. ARG2 deficiency extends lifespan in mice [51], with no significant changes in plasma amino acids and ammonia levels [52]. Therefore, in our study, we aimed to identify potential FDA-approved drugs selective to ARG1 and ARG2 to ameliorate the treatment efficacy.

Virtual screening is a classical tool used to screen novel compounds that target a given protein of interest. Computational screening approaches have gained an advantage over high-throughput screening techniques, due to decreased time and cost to select potential compounds for further experimental validation. In the past, we have used structure-based virtual screening to identify potential ligands for Mcl-1 [53] and Pyk2 [54]. For arginase, several human experimental structures are available and are listed in Table S1.

**Figure 3:**
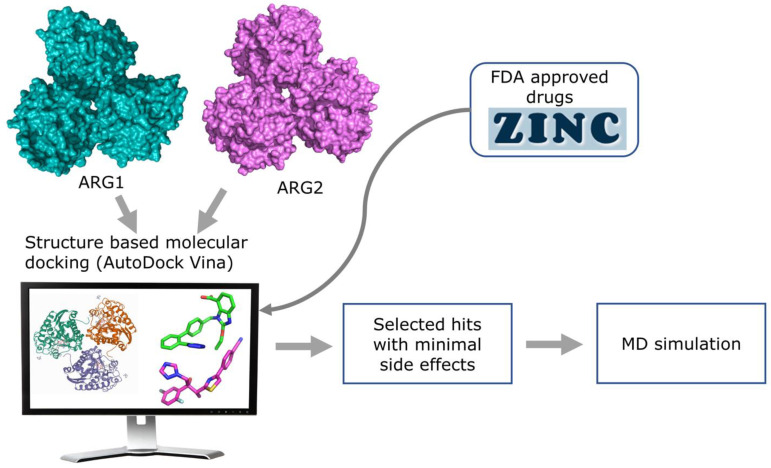
Overview of the structure-based virtual screening workflow.

In this study, we used molecular docking against multiple arginase conformations (3 X-ray crystallography PDB structures for each of the arginase isozymes) to virtually screen for novel ligands that can modulate the activity of arginase, both selectively, and not. In addition, we used molecular dynamics (MD) simulation to validate the protein-ligand stability of the selected hits (Figure 3). Using these techniques with FDA approved drug dataset, we identified novel small molecules that could be repurposed for cancer, Alzheimer’s disease, hypertension, cardiovascular diseases, and other metabolic disorders.

## 2. Materials and Methods

*Database selection and ligand preparation:* FDA-approved drug datasets (DrugBank FDA only, version 2018-02-20) were retrieved in Mol2 format from the non-commercial ZINC15 database (https://zinc.docking.org/ (accessed on 7 February 2022) [55]. We then used the Open Babel package (version 2.3.2) Pittsburgh, PA, USA [56] to convert file format from Mol2 to pdbqt format, followed by Vina Split [57] to obtain 2115 FDA-approved drugs for virtual screening.

*Target preparation and grid generation:* Three High resolution X-ray crystallography structures [PDB IDs 4HWW(1.30 Å) [58], 4HXQ (1.45 Å) [58], 3SJT (1.60 Å) [59]] of ARG1, and three X-ray crystallography structures [PDB ID 4HZE (1.60 Å) [58], 4I06 (1.80 Å) [58], 4IXU (1.90 Å) [60]] of ARG2 were selected among the multiple conformations of these proteins listed in Supplementary Table S1. Protein receptors for virtual screening were prepared further using AutoDockTools (version 1.5.7), California, CA, USA [61], where polar hydrogen atoms were added and Gasteiger charges were assigned. The search space for ligand docking was figured based on the coordinates of the native ligand from the crystal structure. AutoGrid plugin of PyMOL (version 1.8.4.0) New York, NY, USA [62] was used to build a cubic grid box measuring 25 × 25 × 34 Å with 0.375 Å spacing at the binding site of the above-mentioned arginase structures. The X, Y, and Z, coordinates of the center of the docking box were x: −22.48, y: 15.90, z: −12.11 (4HWW); x: −22.64, y: 16.48, z: −11.55 (4HXQ); x: 20.02, y: −13.98, z: 41.4 (3SJT); x: 34.11, y: 87.02, z: 71.22 (4HZE); x: 34.34, y: 86.22, z: 71.47 (4I06); x: 33.34, y: 86.00, z: 72.00 (4IXU).

*In Silico’s high-throughput screening:* AutoDock Vina (1.1.2 for Linux), California, CA, USA [57] was used to run the docking on our Urim high-performance cluster equipped with 64 Intel Xeon processors. In all cases, the default parameters of AutoDock Vina [57] were as follows: The exhaustiveness of the global search was 8, the maximum number of binding modes to generate was 9, and the maximum energy difference between the best and worst binding mode displayed as 3 kcal/moL, thus limiting the number of poses. For each ligand, only the best pose was retained. In all cases, the binding site on arginase was defined and limited by a box measuring 25 × 25 × 34 Å^3^ around different X, Y, and Z, center coordinates.

*Protein-Ligand interaction analysis:* Protein-ligand interaction analysis was performed using PLIP (Protein-Ligand Interaction Profiler) [63] to analyze non-covalent interactions between the arginase and the best docking pose of the selected hit showing fewer side effects.

*MD simulation:* All-atom MD simulation of a protein-ligand complex of ARG1 (PDB ID 3SJT) and ARG2 (PDB ID 4HZE) structures with 5 selected ligands was performed with NAMD (Nanoscale Molecular Dynamics program; version 2.14, Linux-x86_64-multicore-CUDA), Illinois, IL, USA [64] using CHARMM36 forcefield (toppar_c36_jul19.tgz) [65,66,67]. To generate protein parameter files and ligand parameter files VMD (Visual molecular dynamics; version 1.9.3), Illinois, IL, USA [68] and CHARMM-GUI Ligand Reader and Modeler module [69] were used, respectively. As manganese ions were undefined in the CHARMM36 forcefield, we used Mg^2+^ ion parameters instead of Mn^2+^ ions of ARG1 and ARG2, as routinely recommended by Nagagarajan et al. [70]. Solvation and auto-ionization of the protein-ligand complex were performed using VMD. A standard TIP3 water model was added as a solvent with periodic boundary conditions, and a padding distance of 10 Å for each dimension [71]. Na^+^ and Cl^−^ ions were used to neutralize the system. Up to date, the CgenFF version of CHARMM-GUI [72,73] was used to generate topology and parameter files for the selected ligands. Periodic boundary conditions were applied. The particle-mesh Ewald method [74] was used for electrostatic interactions of the system. Minimization was done for 1000 steps at a constant temperature of 298.15 K. Position restraints were applied on Mn^2+^ ion before carrying out equilibration and production run using NPT (constant particle number, pressure, and temperature) ensemble for 50 ns. Three sets of simulations [denoted by Set A (ARG1 (s1a–s5a), ARG2 (s1a–s5a)); Set B (ARG1 (s1b–s5b), ARG2 (s1b–s5b)); Set C (ARG1 (s1c–s5c), ARG2 (s1c–s5c))] were performed for 50 ns each. VMD-Hbonds plugin was used to count strong hydrogen bond (Donor-Acceptor distance 4.0 Å, Angle cutoff 30°) formation between protein and ligand complex throughout the trajectory. RMSD trajectory plugin tool in VMD was used to calculate the RMSD of protein and ligand.

## 3. Results

*Validation of molecular docking assay:* Remarkably, our molecular docking assay was validated using two approaches: (1) First, by comparison of the native crystallographic ligand pose with the top docked pose. Notably, the native and predicted poses yielded good fits with heavy atoms RMSD values for PDB IDs 4HZE (1.47 Å), 4IXU (2.84 Å), 4I06 (2.41 Å), 4HWW (2.12 Å), 3SJT (2.14 Å), and 4HXQ (2.09 Å). (2) Second, by comparison of the experimental dissociation constant (K_d_) of ligands with the predicted one. Notably, AutoDock Vina [57] correctly ranked the following arginase ligands according to their K_d_ 6-nitro-L-norleucine (6HN) (Δ*G_ARG_*_1_ = −5.8, Δ*G_ARG_*_2_ = −6.1), 2(S)-amino-6-boronohexanoic acid (ABH) (Δ*G_ARG_*_1_ = −6.1, Δ*G_ARG_*_2_ = −6.2), nor-N-omega-hydroxy-L-arginine (nor-NOHA) (Δ*G_ARG_*_1_ = −6.2, Δ*G_ARG_*_2_ = −6.3), N-omega-hydroxy-L-arginine NOHA (Δ*G_ARG_*_1_ = −5.9, Δ*G_ARG_*_2_ = −6.5), S-(2-boronoethyl)-L-cysteine BEC (Δ*G_ARG_*_1_ = −6.0, Δ*G_ARG_*_2_ = −6.2) to the various PDB structures of ARG1 and ARG2 used in this study. The predicted K_d_ was calculated from the free binding energy of Vina (ΔG, using the following equation: $K_{d}=e^{\frac{-\Delta G}{RT}}$, and was taken as the average of the top poses in three distinct arginase structures. The predicted K_d_ of ARG1 was taken as the average of the top poses in PDBs 4HZE, 4IXU, and 4I06, while the predicted *K_d_* of ARG2 was calculated using PDBs 4HWW, 3SJT, and 4HXQ. A plot of the predicted and experimental dissociation constant (*K_d_*) is shown in Figure S1. The experimental and predicted *K_d_* correlate well, with correlation coefficients (*R*) of 0.72 for ARG1, and 0.77 for ARG2. These two approaches validate our screening methodology (Table S2). Thus, the predictive power of our in-silico assay was proven in a small dataset and paved the way for further analysis on large datasets.

Less remarkably, the predicted and experimental selectivity of these compounds did not correlate at all. with *R* = 0.0023. The ARG1 selectivity was taken as the ratio of dissociation constant (*K_d_*) of ARG1 divided by ARG2, and the ARG2 selectivity vice versa. The selectivity plot (Figure S2) of experimental and predicted selectivity shows a weak correlation, and as a result, we resorted to using MD simulations for potential selectivity.

*Virtual docking with FDA-approved drug data set:* Virtual docking of 2115 FDA-approved drugs on the six arginase structures, three of ARG1 (PDB IDs: 4HWW, 4HXQ, 3SJT) and three of ARG2 (PDB IDs: 4HZE, 4IXU, 4I06) took ~36 h (Table S3). Notably, the top pose of each ligand is oriented similarly in all three arginases, as indicated by visual inspection, and low RMSDs, (except in less than 2% of the cases) (Table S11). Table 1 lists the top 15 FDA-approved drugs, both non-selective, ARG1 selective, and ARG2 selective. The top 15 non-selective drugs are ranked according to the total of their average binding energy. Interestingly, most of the drugs are used to treat inflammation, headache, cancer, metabolic disorders, and type 2 diabetes which could become clinically relevant for drug repurposing.

Table 1 also lists the top 15 FDA-approved drugs that are potentially selective to ARG1 and ARG2. The difference between the average free binding energies (ΔΔG) of three ARG1 structures and three ARG2 structures is used to rank the top drugs. The top drugs that show potential selectivity for ARG1 are associated with treating fungal and bacterial infection, arthritis, colitis, cancer, depression, and hypertension (Table S4). And the top ARG2 selective drugs are used to treat pain, hypertension, anxiety, infection, heart failure, and malaria among others (Table S4 for indications). Of these, some are associated with severe side effects (i.e., valrubicin, rocuronium, etc.), and are unsafe for repurposing towards arginase inhibition. Some of the ligands were selected based on their relatively low toxicity, and adverse effects. For example, the antihypertensive drugs candesartan, and irbesartan which are angiotensin II receptor blockers (ARB), the thiazide-like diuretic indapamide, the antiemetic rolapitant which is an NK1-receptor antagonist, the anti-angina ivabradine which bind binds to HCN4 receptors, the antihypertensive amiloride that is an epithelial sodium channel (ENaC), the antidiabetic metformin, and the antifungal triazole isavuconazole. Furthermore, metformin was selected because its pharmacokinetic action is not completely understood. These approved drugs have minimal side effects and were further analyzed for their potential repurposing as arginase inhibitors. In particular, we mapped the interaction networks of the selected ligands and performed MD simulations of some with ARG1 (PDB ID 3SJT) and ARG2 (PDB ID 4HZE) structures to check the stability of the protein-ligand interaction.

*Interaction analysis of selected molecules:* The non-selective lead molecules are candesartan (Δ*G_ARG_*_1(3SJT)_ = −8.3, Δ*G_ARG_*_2(4HZE)_ = −8.7), isavuconazole (Δ*G_ARG_*_1(3SJT)_ = −7.1, Δ*G_ARG_*_2(4HZE)_ = −7.0) metformin (Δ*G_ARG_*_1(3SJT)_ = −6.2, Δ*G_ARG_*_2(4HZE)_ = −6.4), amiloride (Δ*G_ARG_*_1(3SJT)_ = −7.8, Δ*G_ARG_*_2(4HZE)_ = −8.2) (Table S5).

**Figure 4:**
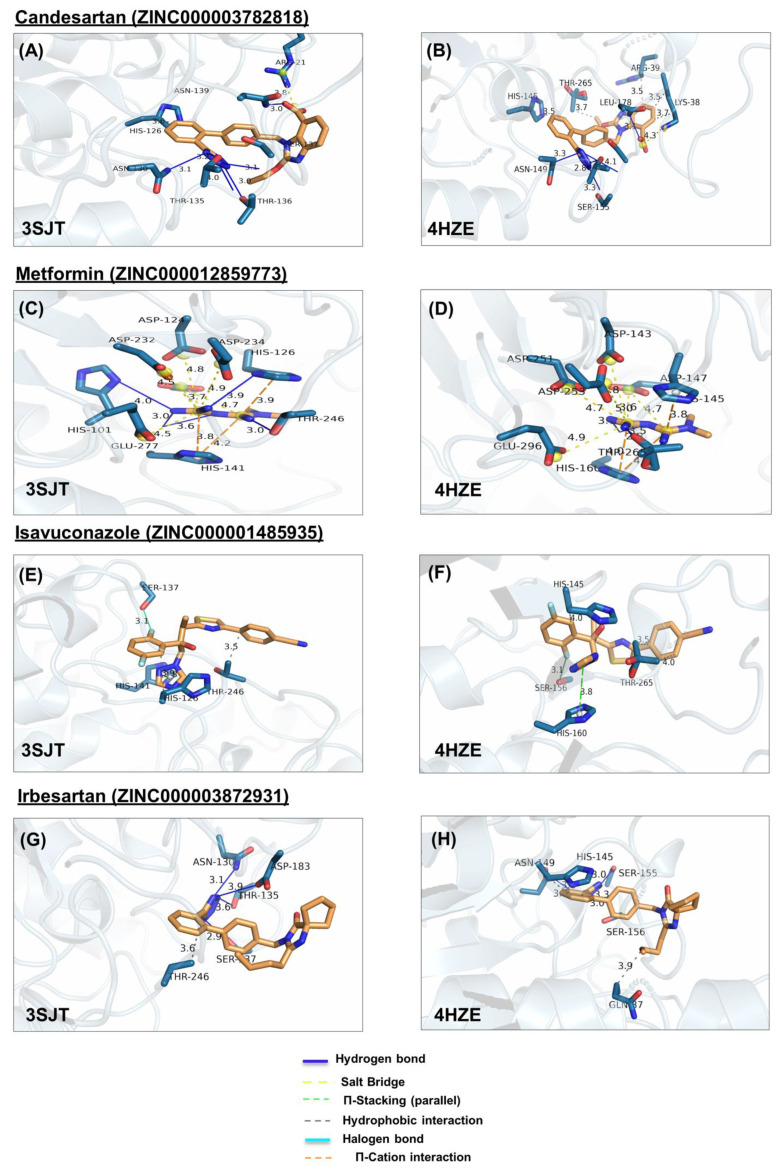

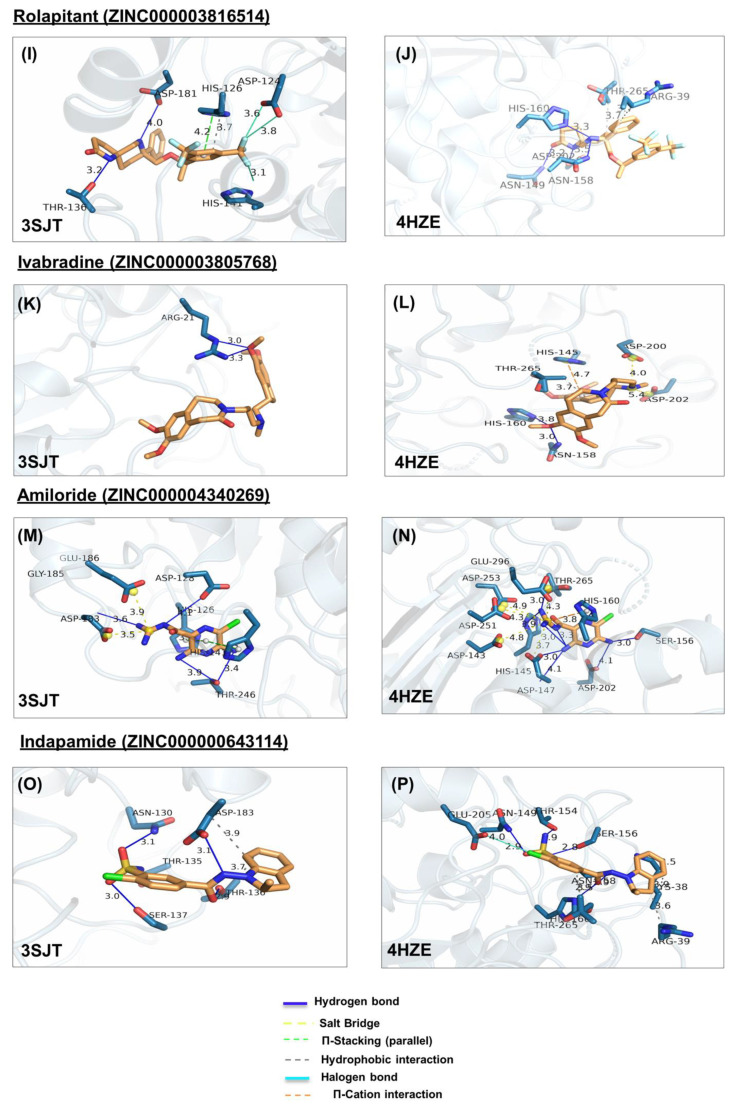
Ligand poses in arginase binding site. (**A**,**B**) Shown is the interaction of the top pose of candesartan with ARG1 and ARG2, (**C**,**D**) interaction with the top pose of metformin. (**E**,**F**) Interaction of the top pose of isavuconazole. (**G**,**H**) Interaction of ARG1 and ARG2 with the best-docked pose of ligand irbesartan. (**I**,**J**) Interaction of best-docked pose of ligand rolapitant with ARG1 and ARG2. (**K**,**L**) Interaction of ARG1 and ARG2 with the best-docked pose of ligand ivabradine. (**M**,**N**) Interaction of best-docked pose of ligand amiloride with ARG1 and ARG2. (**O**,**P**) Interaction of ARG1 and ARG2 with the best-docked pose of ligand indapamide.

In ARG1, metformin forms hydrogen bonds with H101, H126, T246, and H141, salt bridges, with D124, D232, D234, E277, and D128, cation-π with H126 and H141 (Figure 4C). Similarly, in ARG2, it forms hydrogen bonds with H160 and T265, salt bridges with D143, D251, D253, E296 andD147, and cation-π with H145 and H160 (Figure 4D). Note that the numbering of ARG1 and ARG2 residues is shifted by 19 amino acids (i.e., D124 of ARG1 is homologous to D143 of ARG2, etc.)

In ARG1, amiloride forms hydrogen bonds, with D128, D183, and T246, salt bridges with D183, E186, and π-stacking with H126, and H141 (Figure 4M); Similarly, in ARG2, amiloride forms hydrogen bonds with H145, S156, D202, T265, D147, and H160, salt bridges with D143, D147, D251, D253, E296, and cation-π interaction with H160, in ARG2 (Figure 4N).

In ARG1, candesartan forms a hydrophobic interaction with H126, hydrogen bonds with N130, T135, S137, N139, and T136, and salt bridge with R21 (Figure 4A). Similarly, In ARG2, candesartan forms hydrophobic interactions, with R39, H145, T265, and K38, hydrogen bonds with N149, T154, S155, S156, and N158, and salt bridge with K38 (Figure 4B).

In ARG1, isavuconazole forms hydrophobic interactions with H126 and T246, π-stacking with H141, and a halogen bond with S137 (Figure 4E). Similarly, in ARG2, it forms hydrophobic interactions with H145, and T265, π-stacking with H160, and halogen bond with S156 (Figure 4F).

The potential ARG1 selective lead molecule is rolapitant (Δ*G_ARG_*_1(3SJT)_ = −8.4, Δ*G_ARG_*_2(4HZE)_ = −6.8).

In ARG1, rolapitant forms hydrophobic interaction and π-stacking with H126, hydrogen bonds with T136, D181, and halogen bonds with H141 and D124 (Figure 4I). On the other hand, it forms less interaction with ARG2, namely hydrophobic interactions with R39 and T265 and hydrogen bonds with N149, N158, H160, and D202 (Figure 4J).

The potential ARG2 selective lead molecules are irbesartan (Δ*G_ARG_*_1(3SJT)_ = −7.2, Δ*G_ARG_*_2(4HZE)_ = −8.7), indapamide (Δ*G_ARG_*_1(3SJT)_ = −6.7, Δ*G_ARG_*_2(4HZE)_ = −7.9), and ivabradine (Δ*G_ARG_*_1(3SJT)_ = −5.1, Δ*G_ARG_*_2(4HZE)_ = −6.6).

In ARG2, irbesartan forms hydrophobic bonds with Q37, and H145, and hydrogen bonds with S155, N149, and S156 (Figure 4H). In ARG1, it forms weaker interactions, namely hydrophobic interaction with T246, and hydrogen bonds with N130, T135, S137, and D183 (Figure 4G). In ARG2, indapamide forms hydrophobic bonds with R39, T265, and K38, hydrogen bonds with N149, T154, S156, N158, and H160, and salt bridge with E205 (Figure 4P). In ARG1, indapamide form few interactions, namely hydrophobic bonds with T136 and D183, and hydrogen bonds with T135 N130, T136, S137, and D183 (Figure 4O).

In ARG2, ivabradine forms hydrophobic interaction with T265, hydrogen bonds with N158 and HIS160, salt bridges with D200, D202, and π-cation interaction with H145 (Figure 4L). In ARG1 ivabradine forms few interactions, namely a single hydrogen bond with R21 (Figure 4K).

Finally, codeine causes side effects such as addiction, and budesonide is an inhaled glucocorticoid. Nevertheless, preliminary analyses were also performed on them.

Budesonide (Δ*G_ARG_*_1(3SJT)_ = −8.5) forms hydrogen bonds with N130, G142, D183, and salt bridges with H126, and H141 in ARG1 (Figure S3A). Similarly, in ARG2, budesonide (Δ*G_ARG_*_2(4HZE)_ = −8.8) forms a hydrophobic bond with T265, hydrogen bonds with D147, N149, S155, G161, and salt bridges with H145, H160 (Figure S3B).

In ARG2, codeine (Δ*G_ARG_*_2(4HZE)_ = −7.9) forms hydrophobic interaction with T265, hydrogen bonds with S156, N158, H160, T265, and salt bridge with D202 (Figure S3C). Similarly, in ARG1 (Δ*G_ARG_*_1(3SJT)_ = −6.7), it forms a hydrophobic bond with T246, hydrogen bonds with N139, H141, and salt bridges with D181, and D183 (Figure S3D).

**Figure 5:**
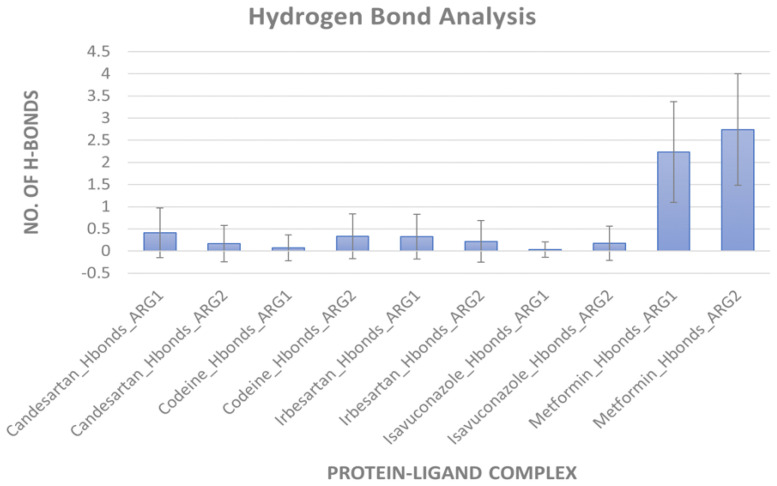
Hydrogen bond formation during MD simulation. An average number of hydrogen bonds between arginases and ligand during 50 ns simulation (Set A). Error bars indicate standard deviation.

**Figure 6:**
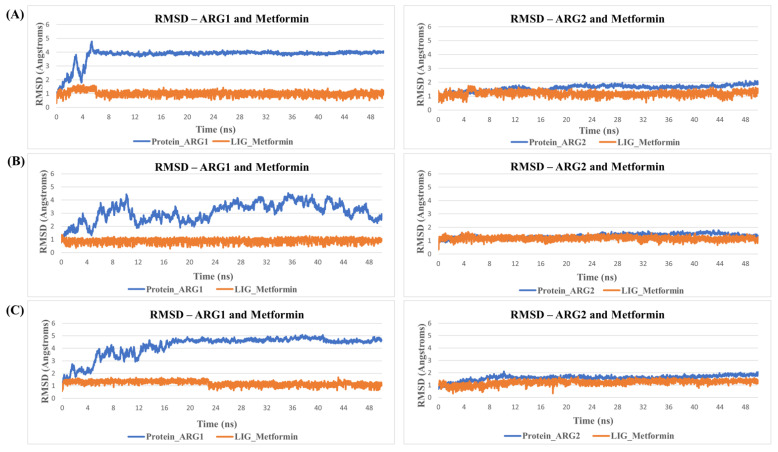
RMSDs during MD simulation. Shown is an example of RMSD values of Metformin in the binding site of arginase during MD simulation. Protein is represented in blue and ligand in orange. Here, (**A**) represent replicate Set A, (**B**) replicate Set B, and (**C**) replicate Set C.

*MD simulation for protein-ligand stability:* Three sets of molecular dynamics simulations up to 50 ns were carried out, to assay ligand interactions and arginase selectivity (candesartan, irbesartan, isavuconazole, codeine, and metformin). Hydrogen bond formation and RMSD analyses of protein-ligand complexes were assessed to evaluate stability (Tables S6–S9). Average RMSD and standard deviation of protein and ligand for each set of the simulation were provided in Table S10. As a control, the binding of the inhibitor nor-NOHA to ARG1 and ARG2 was also simulated [70,75,76]. nor-NOHA remained in the binding site for 50 ns and approx. 16 ns (of 50 ns calculated), respectively (data not shown). Remarkably, candesartan and irbesartan occupied the binding site of ARG1 for the entire length of the simulation (50 ns). In comparison, candesartan and irbesartan remained in the binding site of ARG2 less than 37 ns and 13 ns, respectively, suggesting they are selective for ARG1. This limits, our earlier results which show irbesartan selectivity towards ARG2, and both irbesartan and candesartan could potentially favor ARG1. Interestingly, isavuconazole and metformin remained in the binding site of both arginase for 50 ns, potentially suggesting non-selective arginase binding, thus reinforcing our molecular docking results that isavuconazole and metformin are non-selective. Codeine stayed up to ~8.5 ns with ARG1 before drifting away from the binding pocket to distances obstructive for binding interactions, and 50 ns with ARG2 thus potentially preferring binding to ARG2 in agreement with our molecular docking prediction. Figure 5 and Figure 6 show hydrogen bond formation and RMSD of protein-ligand (Set A) during our MD simulation (Set B and Set C hydrogen bond formation and RMSD of the protein-ligand complex are provided in Tables S6–S10). Thus, our selectivity predictions should be taken with a grain of salt, and require experimental validation. Notably, the amino-rich, metformin forms multiple hydrogen bonds in the binding site with arginase and contributes to complex stability (Figure 5).

## 4. Discussion

ARG1 and ARG2 play a key role in many diseases, such as cancer, Alzheimer’s, hypertension, inflammation, etc. Despite the advances in computer-aided drug design, the clinical trial time frame is a major obstacle to developing a new commercial drug. Thus, using an FDA-approved drug repurposing approach could be advantageous to reduce the clinical trial timeframe and improve treatment. In this study, we used FDA-approved drugs to identify molecules that are (1) non-selective, (2) ARG1 selective, and (3) ARG2 selective. We selected 10 FDA-approved drugs with minimal side effects and studied protein-ligand interaction and stability using a protein-ligand interaction profiler (PLIP) followed by an MD simulation of five selected ligands. We found the antihypertensive drugs candesartan and irbesartan showed potential high affinity towards arginases. Moreover, irbesartan also showed more stability with ARG1 (50 ns) than ARG2 (less than 13 ns) despite having stronger binding energy for ARG2 (Δ*G_ARG_*_1(3SJT)_ = −7.2, Δ*G_ARG_*_2(4HZE)_ = −8.7). We found metformin and isavuconazole are non-selective to both the arginases based on binding energies, protein-ligand interaction, and molecular dynamics. Codeine was more stable with ARG2 (50 ns), which is corroborated by the binding energy (Δ*G_ARG_*_1(3SJT)_ = −6.7, Δ*G_ARG_*_2(4HZE)_ = −7.9). Altogether, candesartan, ibersartan, codeine, metformin, and isavuconazole qualified as suitable candidates for the development of potential arginase inhibitors. Interestingly, irbesartan, and candesartan extend a healthy lifespan in heart failure patients [77], and improve cognitive function in patients [78]. As such, our study exposes potential non-selective, and selective ARG1 and ARG2 ligands to the scientific community for further biological screening, and development of new arginase inhibitors.

As a potential limitation to this study, the AutoDock Vina binding energy is not a quantitative measure of experimental binding energy, however, they are a good measure to qualitatively estimate the relative binding affinity. Like molecular mechanics energies calculated with MM/PBSA and MM/GBSA methods, they are popular approaches to estimating the free binding energy of small ligands to biological macromolecules. Such methods have been applied to a large number of systems with varying success [79]. In this study, we used the AutoDock Vina binding energies to estimate relative binding affinities. Another limitation is our study uses computational methods, which have not been validated experimentally. Thus, additional in-vitro and in-vivo studies are required to characterize ligand binding. Moreover, experimental data and clinical trials are needed to identify the full potential of these selected candidates to serve as arginase inhibitors and improve diseases related to old age, such as hypertension, diabetes, and dementia.

## Figures and Tables

**Table 1:** Top FDA-approved drugs bound to ARG1 and ARG2 conformations.

The Average Binding Energy of FDA-Approved Drugs to ARG1 and ARG2 (kcal/moL)										
Rank	Non-Selective				ARG1_Selective			ARG2_Selective		
	ARG1	SD (±)	ARG2	SD (±)	Ligand	SD (±)	ΔΔG	Ligand	SD (±)	ΔΔG
1	ZINC000003978005Dihydroergotamine (−8.4)	0.1	ZINC000003978005 Dihydroergotamine (−9.2)	0.550757	ZINC000003860453 Fluorescein (−8.3)	0.152753	0.833333	ZINC000043450324 Omacetaxine (−8.8)	0.264575	−1.73333
2	ZINC000169289767 Trypan blue (−8.5)	0.435889894	ZINC000169289767 Trypan blue (−9)	0.971253	ZINC000003816514 Rolapitant (−7.6)	1.078579	0.733333	ZINC000049783788 Valrubicin (−9)	0.251661	−1.6
3	ZINC000004212809 Deflazacort (−8.6)	0.152752523	ZINC000004212809 Deflazacort (−8.7)	0.208167	ZINC000019632668 Doxapram (−6.7)	0.72111	0.7	ZINC000001280665 Hydrocodone (−8.2)	0.152753	−1.6
4	ZINC000052955754 Ergotamine (−8.5)	0.152752523	ZINC000052955754 Ergotamine (−8.8)	0.321455	ZINC000000591993 Nisoldipine (−7)	0.34641	0.666667	ZINC000003872931 Irbesartan (−8.7)	0.11547	−1.6
5	ZINC000064033452 Lumacaftor (−8.4)	0.2081666	ZINC000064033452 Lumacaftor (−8.9)	0.11547	ZINC000003629271 Emtricitabine (−7)	0.83865	0.633333	ZINC000004175630 Pimozide (−8.1)	0.208167	−1.56667
6	ZINC000003927870 Fludarabine (−8.5)	0.1	ZINC000003927870 Fludarabine (−8.8)	0.152753	ZINC000000601250 Sulconazole (−6.6)	0.64291	0.6	ZINC000000000903 Alprazolam (−8.2)	0.152753	−1.56667
7	ZINC000014210876 Eluxadoline (−8.7)	0.1	ZINC000014210876 Eluxadoline (−8.6)	0.2	ZINC000072318121 Abemaciclib (−7.6)	0.23094	0.566667	ZINC000000403533 Oxycodone (−8.5)	0.23094	−1.56667
8	ZINC000004097286 Budesonide (−8.4)	0.2081666	ZINC000004097286 Budesonide (−8.8)	0.057735	ZINC148723177 Brigatinib (−6.4)	0.208167	0.566667	ZINC000001485935 Isavuconazole (−8)	1	−1.53333
9	ZINC000003807172 Argatroban (−8.1)	0.458257569	ZINC000003807172 Argatroban (−8.9)	0.152753	ZINC000100016058 Tipranavir (−7.3)	0.360555	0.533333	ZINC000000001370 Estazolam (−8)	0.057735	−1.53333
10	ZINC000003782818 Candesartan (−8.2)	0.057735027	ZINC000003782818 Candesartan (−8.8)	0.11547	ZINC000000538065 Nefazodone (−6.7)	0.1	0.533333	ZINC000100004343 Artemether (−7.2)	0.208167	−1.5
11	ZINC000006745272 Regorafenib (−8.1)	0.173205081	ZINC000006745272 Regorafenib (−8.9)	0.360555	ZINC000002036848 Riboflavin (−8.1)	0.757188	0.533333	ZINC000003806721 Codeine (−8.1)	0.2	−1.5
12	ZINC000013831130 Raltegravir (−8.2)	0.2081666	ZINC000013831130 Raltegravir (−8.7)	0.1	ZINC000003955219 Darunavir (−7.1)	0.351188	0.533333	ZINC000002019954 Articaine (−7.1)	0.057735	−1.46667
13	ZINC000011679756 Eltrombopag (−8.4)	0.152752523	ZINC000011679756 Eltrombopag (−8.4)	0.057735	ZINC000068204830 Daclatasvir (−7.5)	0.635085	0.5	ZINC000029416466 Saquinavir (−7.6)	0.351188	−1.4
14	ZINC000009212427 Leucovorin (−8.3)	0.288675135	ZINC000009212427 Leucovorin (−8.4)	0.23094	ZINC000000000850 Zileutin (−6.4)	1.101514	0.466667	ZINC000003805768 Ivabradine (−6.4)	0.152753	−1.4
15	ZINC000006716957 Nilotinib (−8.3)	0.152752523	ZINC000006716957 Nilotinib (−8.4)	0.11547	ZINC000022016981 Calteridol (−6.2)	0.360555	0.466667	ZINC000053229445 Rocuronium (−7.1)	0.305505	−1.36667

## Data Availability

Not applicable.

## Supplementary Materials

The following supporting information can be downloaded at: https://www.mdpi.com/article/10.3390/molecules27165134/s1, Figure S1: Correlation plot of experimental and predicted dissociation constant (K_D_). (**A**,**B**) Shown is correlation a plot of experimental and predicted dissociation constant (K_d_) of known arginase inhibitors of ARG1 and ARG2. Notably, the predicted and experimental values correlate well and attest to the accuracy of our molecular docking simulations; Figure S2: Predicted and experimental selectivity. Shown is a plot of the predicted and experimental selectivity of known arginase inhibitors of ARG1 and ARG2; Figure S3: Protein-ligand interactions. (**A**, **B**) Interaction of best docked pose of ligand Budesonide with ARG1 and ARG2. (**C**,**D**) Interaction of best docked pose of ligand Codeine with ARG1 and ARG2; Table S1: Human arginase structures found in the PDB; Table S2: Validation of docking; Table S3: List of FDA approved drugs with their binding energies for PDB structures of ARG1 and ARG2; Table S4: List of top drug candidates—non selective, selective to ARG1 and selective to ARG2; Table S5: Number of intermolecular interactions of the selected drugs; Table S6: Average number of hydrogen bonds during MD simulation (for Set A, Set B, Set C); Table S7: RMSDs of protein ligand complex during MD simulation (Set A); Table S8: RMSDs of protein ligand complex during MD simulation (Set B); Table S9: RMSDs of protein ligand complex during MD simulation (Set C); Table S10: Average RMSD and standard deviation of protein and ligand for each set of simulation; Table S11: RMSDs of top pose of selected ligands in all three PDB structures of each arginase.

## Sample Availability

Samples of the compounds are not available from the authors.

## Abbreviations

FDA—Food and Drug Administration; PDB—Protein data bank, ARG1—arginase type 1, ARG2—arginase type 2, 6HN—6-nitro-L-norleucine, NOHA—N-omega-hydroxy-L-arginine, nor-NOHA—nor-N-omega-hydroxy-L-arginine, ABH—2(S)-amino-6-boronohexanoic acid, BEC—S-(2-boronoethyl)-L-cysteine, NAMD—Nanoscale Molecular Dynamics program, VMD—Visual molecular dynamics, AD—Alzheimer’s disease, PLIP—Protein-ligand interaction profiler, MD Simulation—Molecular dynamics simulation.
