# Supplementary material for: Virtual Screening for FDA-Approved Drugs That Selectively Inhibit Arginase Type 1 and 2
Source: Molecules. 2022 Aug 12;27(16):5134. doi: 10.3390/molecules27165134 (PMC9416497; doi:10.3390/molecules27165134)
Supplement: Supplementary file 1 [file molecules-27-05134-s001.zip › Supplementary_information_legends.pdf]

Supplementary Materials

Figure S1. Correlation plot of experimental and predicted dissociation constant (K_D). A) and B) Shown is correlation a plot of experimental and predicted dissociation constant (K_d) of known arginase inhibitors of ARG1 and ARG2. Notably, the predicted and experimental values correlate well and attest to the accuracy of our molecular docking simulations.

Figure S1. Predicted and experimental selectivity. Shown is a plot of the predicted and experimental selectivity of known arginase inhibitors of ARG1 and ARG2.

Figure S2. Protein-ligand interactions. (A and B) Interaction of best docked pose of ligand Budesonide with ARG1 and ARG2. (C and D) Interaction of best docked pose of ligand Codeine with ARG1 and ARG2.

Table S1. Human arginase structures found in the PDB.

Table S2. Validation of docking.

Table S3. List of FDA approved drugs with their binding energies for PDB structures of ARG1 and ARG2.

Table S4. List of top drug candidates - non selective, selective to ARG1 and selective to ARG2.

Table S5. Number of intermolecular interactions of the selected drugs.

Table S6. Average number of hydrogen bonds during MD simulation (for Set A, Set B, Set C).

Table S7. RMSDs of protein ligand complex during MD simulation (Set A).

Table S8. RMSDs of protein ligand complex during MD simulation (Set B).

Table S9. RMSDs of protein ligand complex during MD simulation (Set C).

Table S10. Average RMSD and standard deviation of protein and ligand for each set of simulation.

Table S11. RMSDs of top pose of selected ligands in all three PDB structures of each arginase.
