# Supplementary material for: Virtual Screening for FDA-Approved Drugs That Selectively Inhibit Arginase Type 1 and 2
Source: Molecules. 2022 Aug 12;27(16):5134. doi: 10.3390/molecules27165134 (PMC9416497; doi:10.3390/molecules27165134)
Supplement: Supplementary file 1 [file molecules-27-05134-s001.zip › Table S1.pdf]

Supplementary Table S1. Human arginase structures found in the PDB.

	PDB ID
Arginase1	1WVA, 1WVB, 2AEB, 2PHA, 2PHO, 2PLL, 2ZAV, 3DJ8, 3E6K, 3E6V, 3F80, 3GMZ, 3GN0, 3KV2, 3LP4, 3LP7, 3MFV, 3MFW, 3MJL, 3SJT, 3SKK, 3TF3, 3TH7, 3THE, 3THH, 3THJ, 4FCI, 4FCK, 4GSM, 4GSV, 4GSZ, 4GWC, 4GWD, 4HWW, 4HXQ, 4IE1,<br>6Q92, 6Q9P, 6QAF, 6V7C, 6V7D, 6V7E, 6V7F
Arginase2	1PQ3, 4HZE, 4I06, 4IE2, 4IE3, 4IXU, 4IXV, 6Q37, 6Q39, 6SS2, 6SS4, 6SS6, 6SRV, 6SRX, 6SS0, 6SS5, 6TUL
