# Supplementary figures and images for: Virtual Screening for FDA-Approved Drugs That Selectively Inhibit Arginase Type 1 and 2
Source: Molecules. 2022 Aug 12;27(16):5134. doi: 10.3390/molecules27165134 (PMC9416497; doi:10.3390/molecules27165134)

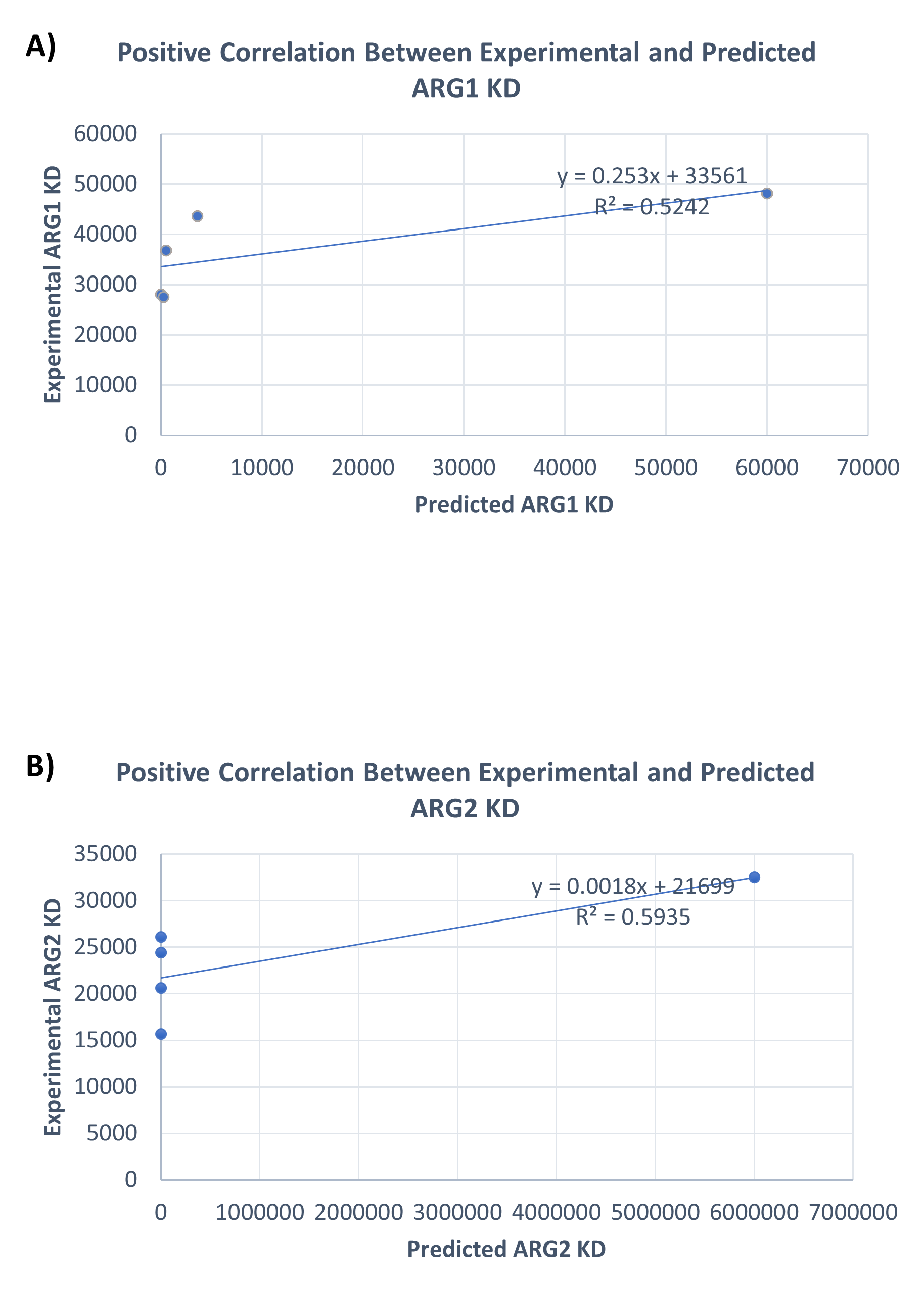

Supplement: Supplementary file 1 [file molecules-27-05134-s001.zip › Figure S1.png]

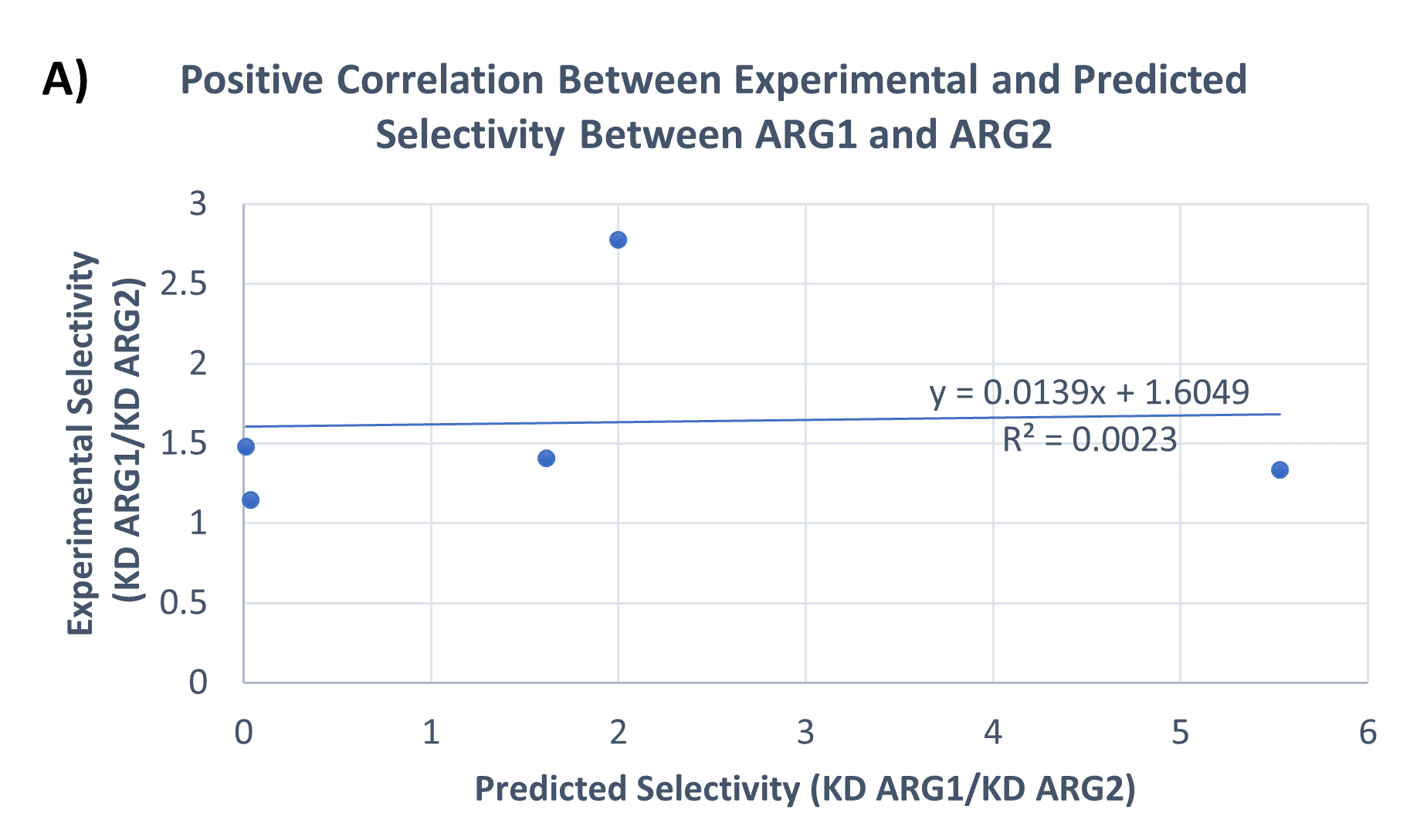

Supplement: Supplementary file 1 [file molecules-27-05134-s001.zip › Figure S2.png]

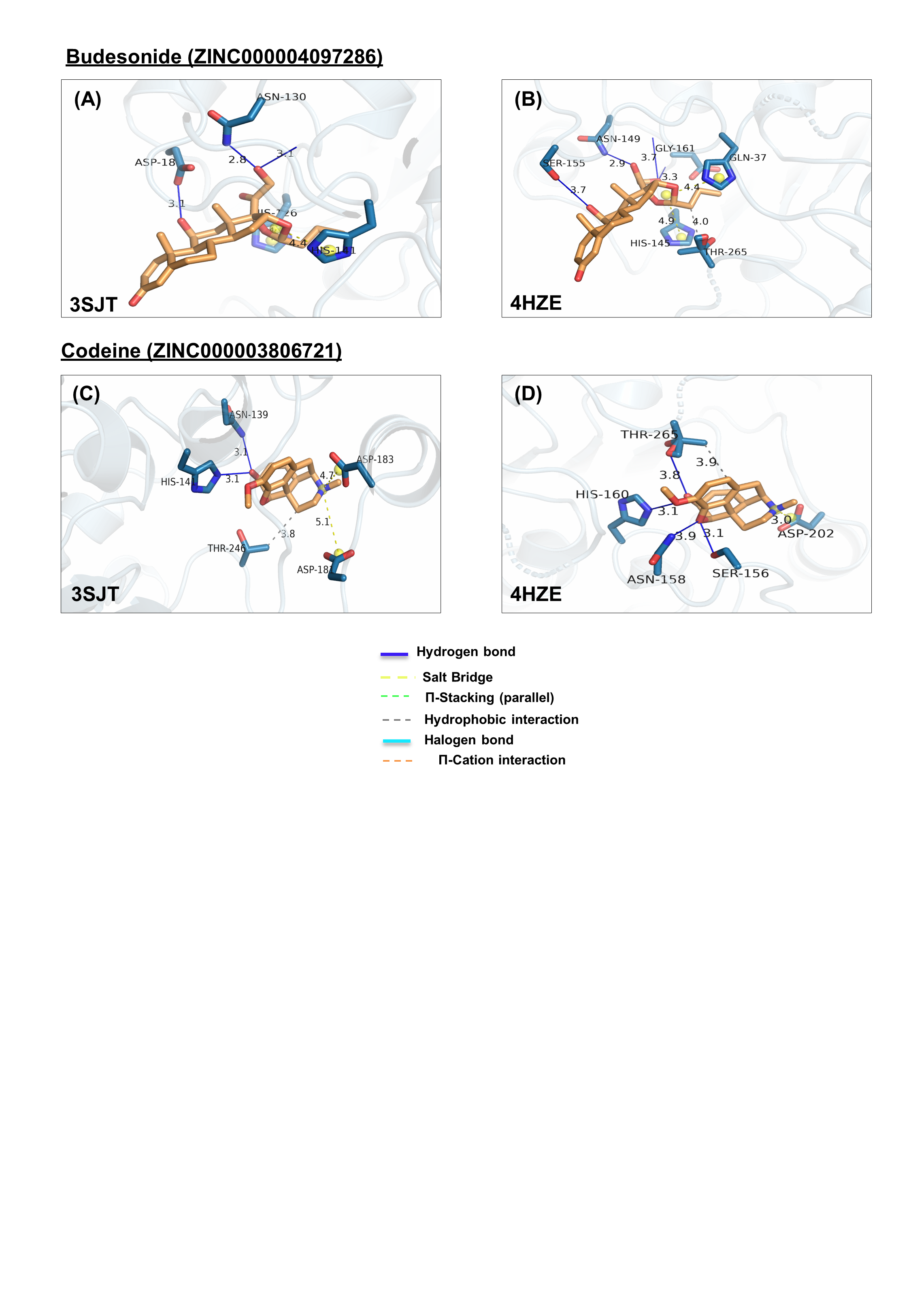

Supplement: Supplementary file 1 [file molecules-27-05134-s001.zip › Figure S3.png]
